# A rare case of sinonasal glomangiopericytoma post operative accidental diagnosis and managment—A case report

**DOI:** 10.1016/j.ijscr.2019.06.066

**Published:** 2019-08-09

**Authors:** Nitin Sharma, Dushyant Mandlik, Purvi Patel, Parin Patel, Aditya Joshipura, Mitesh Patel, Srinal Mankiwala, Ashutosh Vatsyayan, Tulika Dubey, Kintan Sanghvi, Diva Shah, Shubhada Kanhere, Shailesh Talati, Kaustubh Patel

**Affiliations:** a HCG Cancer Center, Sola, Ahmedabad, Gujarat, India; b Hemato Oncology Clinic, Ahmedabad, Gujarat, India

**Keywords:** Glomangiopericytoma, Haemangiopericytoma, Nasal, Tumor, Endoscopic sinus surgery

## Abstract

**Introduction:**

Glomangiopericytoma is a rare neoplasm of low malignant potential. It is a rare type of haemangiopericytoma located in nasal cavity. This neoplasm has good prognosis and complete surgical excision is treatment of choice. This case report is representing one such neoplasm. This reporting is done in line with the SCARE criteria (Agha et al., 2018 [1]).

**Case presentation:**

We presenting a case of 54 year old male patient from upper socioeconomic status who presented at our institution with history of surgery (endoscopic sinus surgery with Septoplasty) 15 days back. A Final histopathology report suggested glomangiopericytoma. Since primary surgery was not done as per oncologic principals, patient was advised for adjuvant radiation. At our institute patient was evaluated again. Revision surgery was done. Patient was discharged next day.

**Discussion:**

Glomangiopericytoma is a rare neoplasm with incidence of less than 0.5% of all neoplasms of sinonasal cavity. Prognosis is very good after complete surgical excision. It often confuses clinicians with nasal polyps. Here also patient was operated initially considering as benign polyposis outside. Patient was re-operated again to ensure the complete clearance.

**Conclusion:**

This is the typical case of converting dual modality treatment to single modality with the help of knowledge, communication, transparent team work. This also a rare type of neoplasm and by reporting this rare case we are contributing to data pool of nasal tumors where lack of reporting is major obstacle in the formation of uniform treatment guidelines.

## Highlights

• Glomangiopericytoma is rare sinonasal disease with very good prognosis after surgical excision.
• Prognosis is very good after surgical excision.
• As most nasal tumors our case was also an accidental diagnosis following endoscopic sinus surgery. and complete clearance was suspicious.
• Patent was referred for adjuvant radiation.
• Revision surgery was done and wide clearance was established.
• Patient was saved from radiation.

## 1 Case

**Fig. 1:**
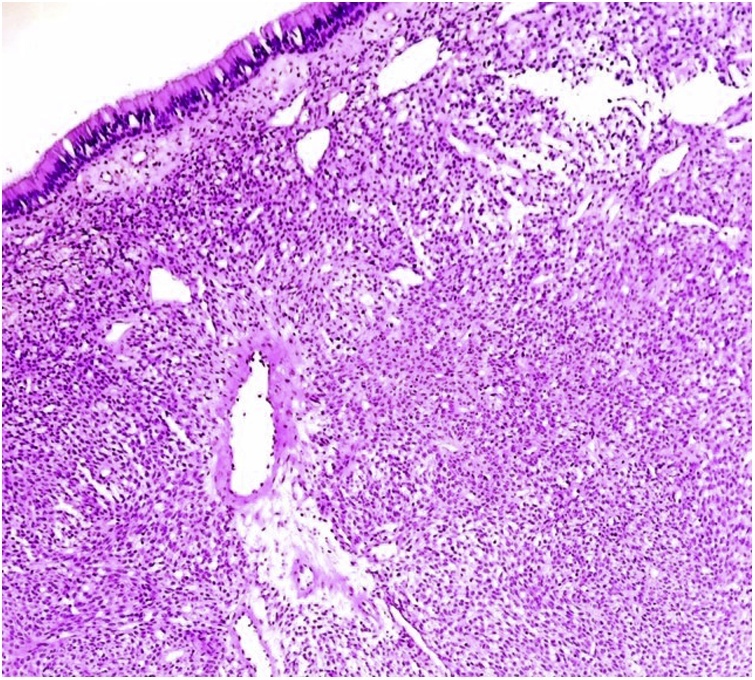
Respiratory epithelium and subepithelial sheets of oval tumor cells arrange themselves around blood vessels. (H&E X 40).

**Fig. 2:**
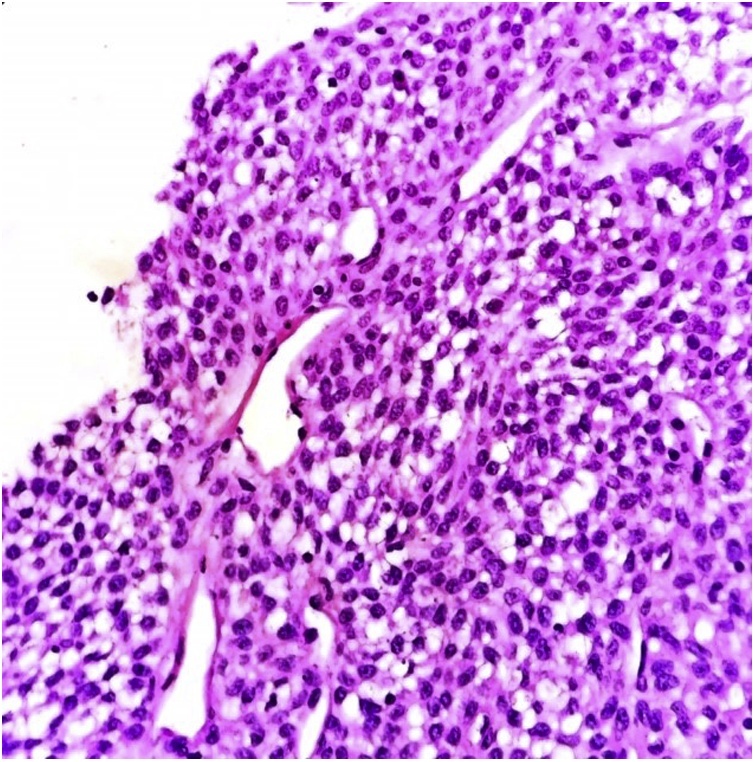
Oval tumor cells around blood vessels show very minimal atypia. (H&E X 100).

This is a case of 65 year old male who presented in outpatient department in tertiary care institute. He was known case of bronchial asthma and had history of myocardial infarction seven years back. Presently he was on anti-platelet medication. Patient was operated outside for septoplasty and functional endoscopic sinus surgery (FESS) 25 days back. Patient was referred by medical oncologist for surgical opinion about further management. Presenting symptoms were pain in nasal cavity with occasional bloody discharge. The previous surgery was done by otolaryngologist. CT scan done prior to the first surgery was reported as polypoidal mucosal thickening in sphenoid sinus with complete opacification. There was hyperdensity without any abnormal enhancement but mild focal extension of soft tissue was seen in sphenoethmoidal recess left side and protruding into nasopharynx. There was neither erosion of seller floor nor intracranial extension. No histopathology report was done prior to the surgery. The final histopathology report after the surgery was reported as respiratory mucosa with a submucosal tumor with plasmacytoid and focally spindled morphology in myxoid to vascularized stroma and differential diagnosis was given as minor salivary gland neoplasm, nasal tumor and extraosseous myeloma (Fig. 1, Fig. 2).

**Fig. 3:**
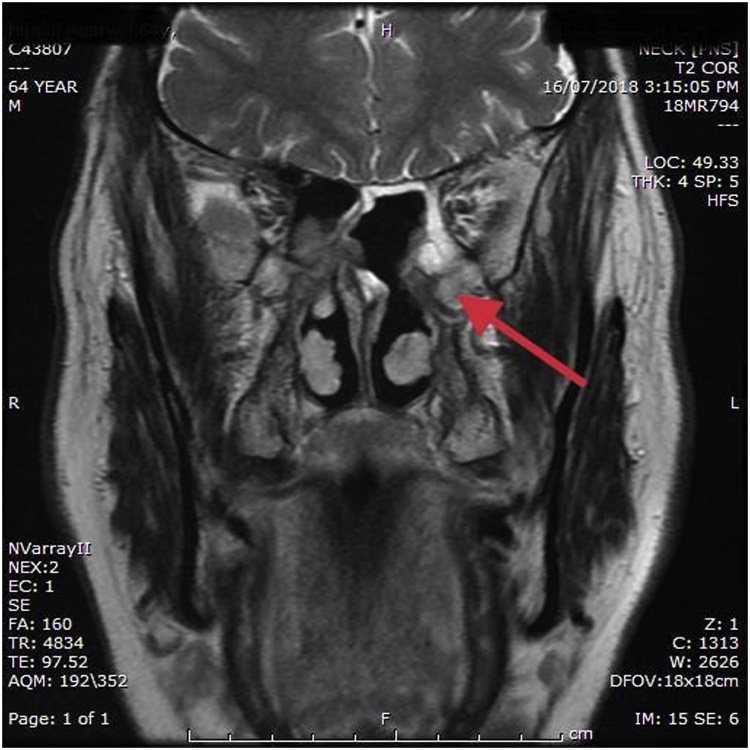
Post first surgery magnetic resonance imaging with contrast coronal section of paranasal sinus.

**Fig. 4:**
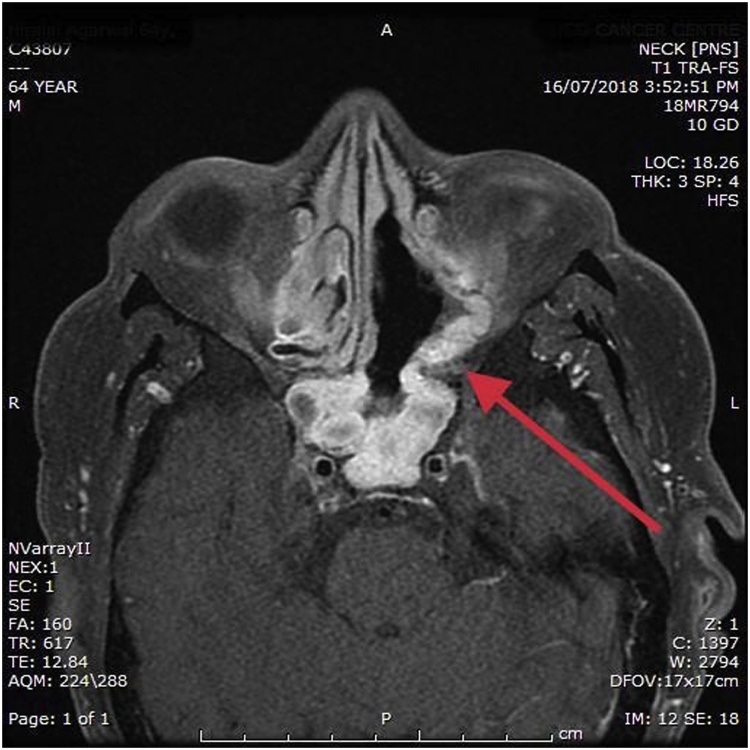
Post first surgery magnetic resonance imaging with contrast axial section of paranasal sinus.

**Fig. 5:**
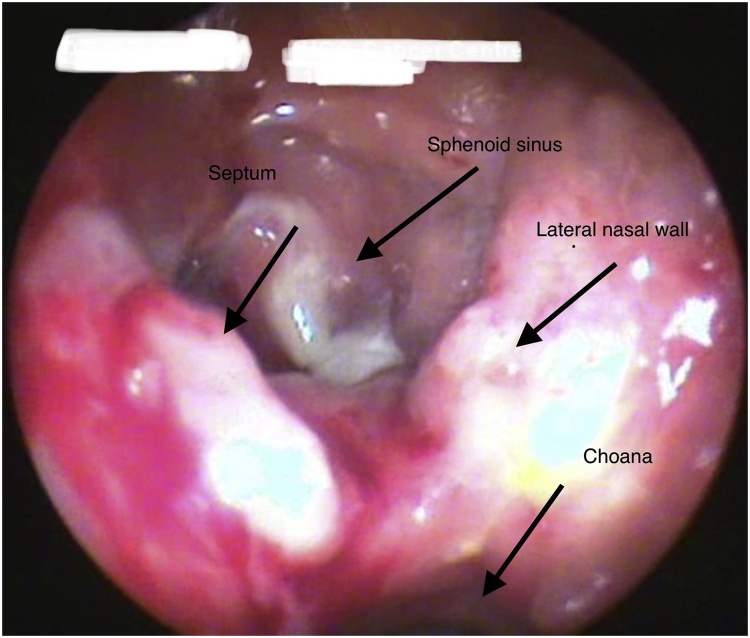
Endoscopic image after first surgery and before second (revision) surgery showing granulation tissue at posterior part of septum, floor of sphenoid sinus and adjacent lateral nasal wall.

His Immunohistocytochemistry (IHC) was reported positive for SMA but negative for desmin,CD-34, S-100 protein, cytokeratin and EMA and concluded as glomangiopericytoma. Immunohisto cytochemistry (IHC) was done at one more place where tumor was reported positive for SMA and beta catenin but negative for synaptophysin, P40, P63, S100, CK7, MUM1, DESMIN, CD 99, CD34 and CD31 and concluded as borderline/low grade malignant soft tissue tumor: sinonasal glomangiopericytoma. After this report patient was referred to the radiation oncologist for adjuvant radiation as primary surgeon was not sure of complete excision. Through a common medical oncologist friend patient was referred to us. At our centre on examination we found operated cavity full of granulation tissue. It was difficult to establish complete surgical excision clinically which was very important in this case. Patient underwent MR scan to rule out the presence of any residual disease. The MRI was reported as ill defined low signal intensity areas in posterior- superior aspect of left nasal cavity and sphenoethmoidal recess most likely the postoperative granulation tissue/fibrosis. Still they advised for clinical correlation (Fig. 3, Fig. 4, Fig. 5).

Now the challenges were.

1 We wanted to avoid radiation but that can be done only after the establishment of complete clearance.
2 Radiologists were not sure about complete excision.
3 It was very difficult to differentiate between the residual disease and postoperative granulation tissue clinically.
4 One opinion was to wait till granulation tissue heals but in that case patient was losing the golden time for commencement of radiation.
5 Going again in recently operated cavity is itself challenge but only complete clearance offers the best chance of cure.

All these issues were discussed in tumor board where it was decided to establish that there was no residual tumor. This case was also discussed with the patient and his son. After through discussion and fully informed consent it was decided to go for revision surgery with frozen section to achieve 360° clearance.

As per plan patient was operated again endoscopically and entire granulation tissue was excised and subjected to frozen section along with normal mucosal margins. Frozen section was reported negative for residual disease. Entire hospital stay was uneventful and patient was discharged on second postoperative day.

**Fig. 6:**
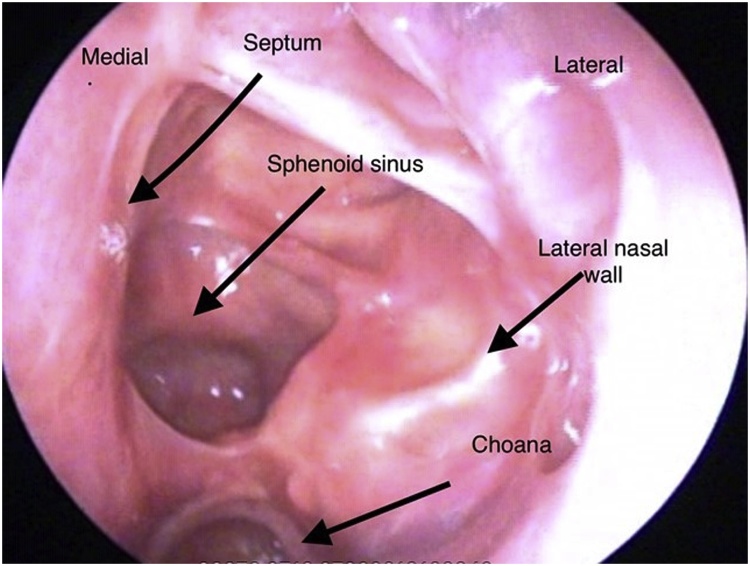
Endoscopic image after one year (revision surgery) showing well healed mucosa in nasal cavity at posterior part of septum, floor of sphenoid sinus, and adjacent lateral nasal wall.

Final histopathology report was negative for residual disease and all the margins and tumor bed mucosa was free from tumor. Patient was now advised for observation instead on adjuvant radiation. Since then patient is in regular follow with for last 9 months with us and he is disease free. Patient had also underwent post second surgery MRI which was reported negative for disease (Fig. 6).

## 2 Discussion

Any mass in a nasal cavity is very thrilling for endoscopic surgeon. Mild symptoms and causal approach of both clinicians and patient create room for surprises. Most of the time unilateral soft, insensitive and grayish mass is nasal polyp but not always. Here we present a case where a nasal mass with same characteristics was operated by a endoscopic surgeon considering this as sinonasal polyp and later in final histopathology report it turned out as glomangiopericytoma. The incidence of glomangiopericytoma is less than 0.5% of all sinonasal tumors [2]. Glomangiopericytoma as name suggests originates from pericytes [2].

Pericytes are cells in the walls of capillaries [3]. Glomangiopericytoma behaves as indolent tumor and confuses the clinicians due to appearance similar to inflammatory polyps [4]. The first case was reported in 1942 by Staut and Murray and was classified as hemangiopericytoma [5].

In 1905 WHO recognised this as a separate entity [4]. As initial surgery was done considering lesion as inflammatory polyp. Although it was done with computed tomography of paranasal sinus but without contrast study and without prior biopsy of nasal mass. No intraoperative frozen section was sent. Patient and relatives were not explained about possibility of lesion being malignant on final histopathology and a second wide excision or adjuvant treatment. Final histopathology and immunohistochemistry turned out surprise as glomangiopericytoma. Treatment of choice for glomangiopericytoma is complete surgical excision of tumor [6] and adjuvant treatment is generally required if tumor is excised incompletely. In series of 104 patient Thompson has reported survival rate of 74.2 percent at 5 years with recurrence rate is 17 percent [7]. In this case neither tumor margins nor complete excision was sure that is why adjuvant treatment was advised by treating surgeon. To avoid the adjuvant treatment and morbidity associated with it with the consent of the patient revision surgery was done. In second surgery complete excision with clear margins was ensured. Thus we saved the patient from adjuvant treatment and it’s morbidity. There are several reasons for is to report this case.

On reviewing literature we did find too many cases of glomangiopericytoma. Many more case reports are required to generate adequate data pool.

This is the example of teamwork where the final goal of the team was to do best for the patient and we did it.

So for us the take home message from this case is that there should be a comprehensive care for any tumor then only we can provide best to the patient.

## 3 Conclusion

Glomangiopericytoma is rare indolent kind of neoplasm. The treatment of choice for glomangiopericytoma is surgery. The best can only be achieved if you work in team. More and more reporting of cases will help in making definitive guidelines for the treatment for glomangiopericytoma.

## Funding

We wish to confirm that there has been no significant financial support for this work that could have influenced its outcome.

## Ethical approval

Not applicable for this case report.

## Consent

I wish to confirm that the written and informed consent from the patient has been taken from the patient to publish this case report.

## Author contribution

Dr. Nitin Sharma- performed the surgery. Prepare the case report.

Dr. Dushyant Mandlik - communication with the patient and preparation of case report.

Dr. Purvi Patel - post operative care.

Dr. Parin Patel - planning.

Dr. Aditya Joshipura-post operative care.

Dr. Mitesh Patel - post operative care.

Dr. Srinal Mankiwala - assistant surgeon.

Dr. Ashutosh Vatsayayan - assistant surgeon.

Dr. Tulika Dubey- arranging pathology pictures.

Dr. Kintan Sanghvi - pathology report and pictures.

Dr. Diva Shah - radiology work up and pictures.

Dr. Shubhada Kanhere - pathology pictures.

Dr. Shailesh Talati - planing and management.

Dr. Kaustubh Patel - planning the management and concept.

## Registration of research studies

Not applicable for this case report.

## Guarantor

Dr. Dushyant Mandlik.

## Provenance and peer review

Not commissioned, externally peer-reviewed.

## Declaration of Competing Interest

We wish to draw the attention of the Editor to the following facts which may be considered as potential conflicts of interest and to significant financial contributions to this work.
